# Mechanical Durability of Flexible Printed Circuit Boards Containing Thin Coverlays Fabricated with Poly(Amide-Imide-Urethane)/Epoxy Interpenetrating Networks

**DOI:** 10.3390/mi12080943

**Published:** 2021-08-10

**Authors:** Jeongah Kim, Bo-Young Kim, Seong Dae Park, Ji-Hun Seo, Chan-Jae Lee, Myong Jae Yoo, Youngmin Kim

**Affiliations:** 1 Electronic Materials & Device Research Center, Korea Electronics Technology Institute, Seongnam 463-816, Korea; jeongahkim@keti.re.kr (J.K.); kby0827@keti.re.kr (B.-Y.K.); sdpark@keti.re.kr (S.D.P.); 2 Department of Materials Science and Engineering, Korea University, Seoul 136-701, Korea; seojh79@korea.ac.kr; 3 Display Research Center, Korea Electronics Technology Institute, Seongnam 463-816, Korea; chanjael@keti.re.kr

**Keywords:** flexible printed circuit boards, coverlay, poly(amide-imide-urethane), interpenetrating network, MIT folding test

## Abstract

Because electronics are becoming flexible, the demand for techniques to manufacture thin flexible printed circuit boards (FPCBs) has increased. Conventional FPCBs are fabricated by attaching a coverlay film (41 μm) onto copper patterns/polyimide (PI) film to produce the structure of coverlay/Cu patterns/PI film. Given that the conventional coverlay consists of two layers of polyimide film and adhesive, its thickness must be reduced to generate thinner FPCBs. In this study, we fabricated 25-μm-thick poly(amide-imide-urethane)/epoxy interpenetrating networks (IPNs) to replace the thick conventional coverlay. Poly(amide-imide-urethane) (PAIU) was synthesized by reacting isocyanate-capped polyurethane with trimellitic anhydride and then mixed with epoxy resin to produce PAIU/epoxy IPNs after curing. Thanks to the soft segments of polyurethane, the elongation of PAIU/epoxy IPNs increased with increasing PAIU content and reached over 200%. After confirming the excellent thermal stability and chemical resistance of the PAIU/epoxy IPNs, we fabricated FPCBs by equipping them as coverlays. The mechanical durability of the FPCBs was evaluated through an MIT folding test, and the FPCB fabricated with PAIU/ep-2 was stable up to 164 folding cycles because of the balanced mechanical properties.

## 1. Introduction

**Figure 1:**
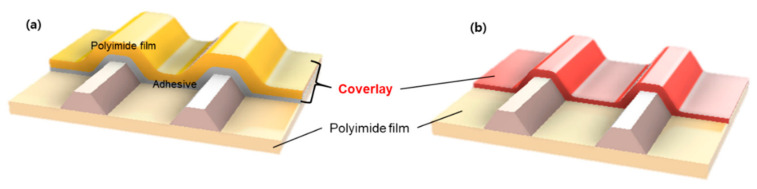
Schematics of the FPCB structure containing (**a**) conventional coverlay (**b**) this work PAIU.

In response to increasing demand for flexible electronics, much effort has been made to fabricate flexible printed circuit boards (FPCBs) in which narrow copper (Cu) metal lines are formed on a flexible polyimide substrate and protected with a coverlay [1]. Given that the stress and strain are concentrated on a small region in wearable electronics during repeated use, the thinner the FPCBs are, the more durable the electronics are. To make FPCBs thinner, the 41-μm-thick coverlay comprising a polyimide film and an epoxy adhesive layer was substituted by a polyimide layer in previous studies [2,3,4]. A schematic diagram of the FPCB structures fabricated through a film (polyimide and adhesive) attachment and a simple coating of polyimide solution is shown in Figure 1. However, because of the low solubility of polyimide resins in organic solvents, their precursors, poly(amic acid)s, were coated and then heated to 300 °C to fabricate polyimide layers [5]. Given that high temperature deteriorates the electrical performance of Cu electrodes, a process that can be carried out at a lower temperature to fabricate a coverlay is needed. Hence, polyamideimide (PAI) has attracted considerable attention due to its high solubility in various organic solvents and has excellent performance stemming from the amide groups in the polymer backbone [6,7,8,9,10,11,12,13,14]. In addition, the process requires a lower temperature (<200 °C) than the imidization of poly(amic acid)s to fabricate PAI layers on circuit boards. In this study, a soft polyurethane was incorporated into the PAI backbone to attain high flexibility of the product (PAIU) [15,16]. The polyurethane was composed of polyether-based soft segments and urethane-based hard segments. While soft segments endow the polyurethane with flexibility, hard segments engaging in hydrogen bonding improve the mechanical properties of the polyurethane. The elastomeric properties of polyurethane are generally ascribed to the phase separation of rigid segments, which serve as crosslinking points, from soft phases [17,18,19,20,21]. In previous studies [22,23,24], elastomers were used to fabricate stretchable electronic devices with excellent mechanical properties. The synthesized PAIU was mixed with epoxy resin to fabricate PAIU/epoxy interpenetrating polymer networks (IPNs) after curing. After investigating the mechanical properties, thermal stability, and chemical resistance of the PAIU/epoxy IPNs, they were used to fabricate FPCBs in which they served as coverlays. The mechanical durability of the FPCBs was evaluated through an MIT folding test.

## 2. Experiments

### 2.1. Materials

4,4′-Diphenylmethane diisocyanate (MDI) (98%), poly(tetrahydrofuran) (PTHF) (Mn = 1000 g/mol), and N-methyl-2-pyrrolidone (NMP) (anhydrous, 99.5%) were purchased from Sigma-Aldrich Korea Ltd. (Yongin, Korea). Trimellitic anhydride (TMA) (97%) and 2,4-diamino-6-[2-(2-methyl-1-imidazolyl)ethyl]-1,3,5-triazine (2MZ-A) (98%) were purchased from Tokyo Chemical Industry Co., Ltd. (Tokyo, Japan). Biphenyl novolac epoxy resin (EEW = 280–300 g/eq, 80 wt% in methyl ethyl ketone) was purchased from SHIN-A T&C, (Hwaseong, Korea). All chemicals were used as received without further purification. A polyethylene terephthalate (PET) bottle was purchased from Daihan Scientific (Wonju, Korea). An acidic solution for chemical resistance test was hydrochloric-acid- and chloric-acid-based copper chloride solution.

### 2.2. Instrumentation

^1^H and ^13^C NMR spectra were measured on a nuclear magnetic resonance (NMR) spectrometer (Ascend 600 MHz, Bruker, Madison, WI, USA). The Fourier transform infrared (FTIR) spectra were obtained by the attenuated total reflectance method using an FTIR spectrophotometer (Nicolet 6700, Thermo Fisher Scientific, Waltham, MA, USA). The molecular weight and the distribution were detected by gel permeation chromatography (GPC, Young In Chromass, Anyang, Korea) using a YL9112 pump and a YL9170 refractive index detector with tetrahydrofuran (THF) as the mobile phase with a flow rate of 1.0 mL/min and polystyrene (PS) as the standard. Mechanical properties of the specimens were measured using a universal testing machine (UTM) (5543, Instron, Norwood, MA, USA). Thermo mechanical properties of the samples were evaluated by dynamic mechanical analysis (Q800, Waters Corporation, MA, USA) from 30 to 300 °C with a frequency of 1 Hz and a heating rate of 3 °C/min. Thermal decomposition was verified using thermogravimetric analysis (SDT Q600, Waters Corporation, MA, USA). An MIT folding endurance test was carried out using an MIT-DA instrument (TOYO Seiki, Chino, Japan).

### 2.3. Synthesis of Poly(Amide-Imide-Urethane) (PAIU)

A 500-mL three-neck flask equipped with a thermometer, a reflux condenser, and an N_2_ gas inlet was charged with PTHF (38.04 g, 0.04 mol), MDI (19.04 g, 0.08 mol), and NMP (83.13 mL). The solution was stirred at room temperature and then slowly heated to 80 °C. After 3 h of stirring, the flask was allowed to cool to room temperature and TMA (7.31 g, 0.04 mol) was added. The mixture was heated at 75 °C for 30 min and 125 °C for 2 h, sequentially. It was then cooled to room temperature to prepare the solution containing PAIU. The solution was added to an ethanol/acetone mixture (80 wt%/20 wt%), and the precipitate was filtered, followed by drying in an oven to afford a yellow solid as PAIU.

### 2.4. Preparation PAIU/Epoxy Blends

The compositions of the PAIU/epoxy blends are summarized in the Table 1. The as-prepared PAIU, an epoxy solution (a solid content of 80 wt%), and a curing agent were added to a polyethylene phthalate (PET) bottle and mixed by ball-milling at 30 rpm for 10 h.

### 2.5. Preparation of Test Specimens

The as-prepared PAIU/epoxy blends were poured into a silicone mold with dimensions of 125 mm × 25 mm on a Teflon sheet which was on a glass substrate, heated at 150 °C for 2 h and at 180 °C for 70 min sequentially to produce the PAIU/epoxy IPN films. For a tensile test, dog-bone-shaped samples were obtained by cutting the films with a pressing mold. In addition, the 12 mm × 8 mm specimens for dynamic mechanical analysis were prepared by cutting the films using a CO_2_ laser.

### 2.6. Fabrication of Flexible Printed Circuit Boards (FPCBs)

Each PAIU/epoxy blend was bar-coated onto a printed circuit board with the structure of Cu patterns/PI film (18 mm × 140 mm) and cured at 150 °C for 2 h and 180 °C for 70 min sequentially to produce the structure of PAIU/epoxy IPNs (25 μm)/Cu patterns/PI film in which the PAIU/epoxy IPNs serves as coverlays.

### 2.7. MIT Folding Endurance Test

The as-prepared FPCBs were subject to an MIT folding test with a rotation angle of ±135°, a radius of 0.38 mm, a folding speed of 175 rpm, and a weight loading of 250 g. The number of times each specimen was folded was recorded until the Cu patterns were ruptured [25,26].

## 3. Results and Discussion

**Scheme 1:**
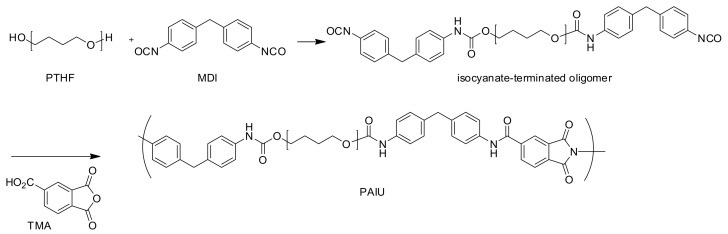
Synthesis of an isocyanate-terminated oligomer and PAIU.

**Figure 2:**
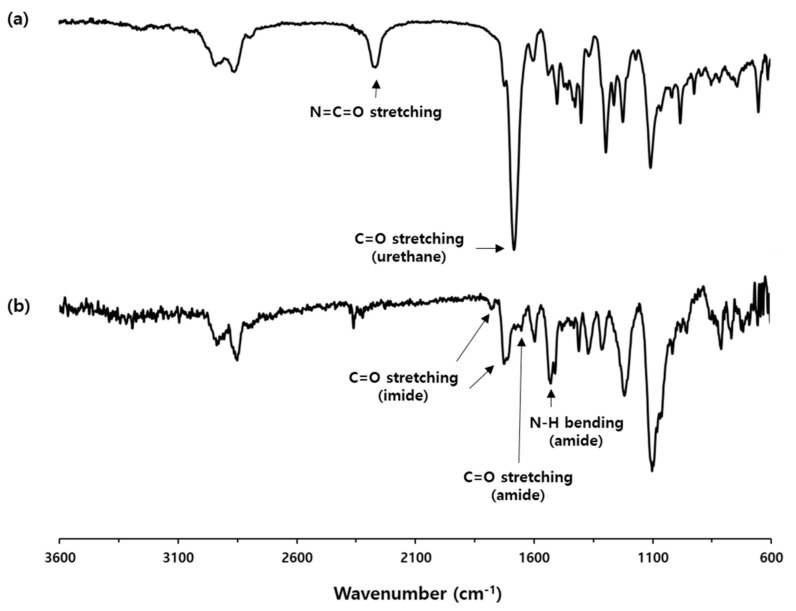
FTIR spectra of (**a**) an isocyanate-terminated oligomer and (**b**) PAIU.

In this study, coverlays for FPCBs were fabricated by curing poly(amide-imide-urethane)/epoxy blends. Given that an isocyanate group reacts with anhydride and carboxylic acid to produce imide and amide, respectively, soft isocyanate-capped oligomers are expected to react with trimellitic anhydride (TMA) to produce poly(amide-imide-urethane)s (PAIUs) (Scheme 1). Therefore, poly(tetrahydrofuran) was chosen as a soft segment and allowed to react with 4,4′-diphenylmethane diisocyanate at an OH-to-NCO molar ratio of 1-to-2, which produced an isocyanate-terminated oligomer. The viscosity of the solution was measured as 230 cps at 25 °C. FTIR spectroscopy was conducted to characterize the oligomer. In the FTIR spectrum of the oligomer, intense IR absorption was observed at 1684 cm^−1^ for C=O stretching confirming the formation of urethane bonds (Figure 2a). The presence of isocyanate groups was proved by the IR absorption at 2267 cm^−1^ as well [27,28,29,30,31,32,33]. The number-average (Mn) and weight-average (Mw) molecular weights of the oligomer were measured to be 5 and 9 kg/mol, respectively, with a polydispersity index (PDI) of 1.69.

Next, this oligomer was allowed to react with TMA to produce soft PAIU, as shown in Scheme 1. The completion of the reaction was confirmed by the disappearance of the IR absorption for N=C=O stretching (Figure 2). The viscosity of the solution increased to 9300 cps at 25 °C, indicating that the polymerization reaction had occurred. After the product was isolated from a NMP/ethanol/acetone mixture, it was characterized by FTIR and NMR spectroscopy. The formation of amide bonds was ascertained by the intense IR absorption at 1652 and 1530 cm^−1^ corresponding to C=O stretching and N–H bending, respectively (Figure 2b). The strong IR peaks at 1727 and 1778 cm^−1^ indicate the formation of imide bonds [34,35,36]. In the ^1^H NMR spectrum of PAIU, the proton signals for aryl groups of diphenylmethane and phthalimide appear in the ranges from 7.14 to 7.57 ppm and from 8.54 to 8.59 ppm, respectively. The proton peaks resonating from 9.47 to 10.73 ppm correspond to (CO)NH of amide and urethane groups. The presence of urethane, amide, and imide groups was further supported by carbon signals at 153.4, 164.7, and 167.1 ppm in the ^13^C NMR spectrum of PAIU [37,38]. The Mn and Mw of PAIU were determined to be 19 and 36 kg/mol, respectively, with a PDI of 1.91.

**Figure 3:**
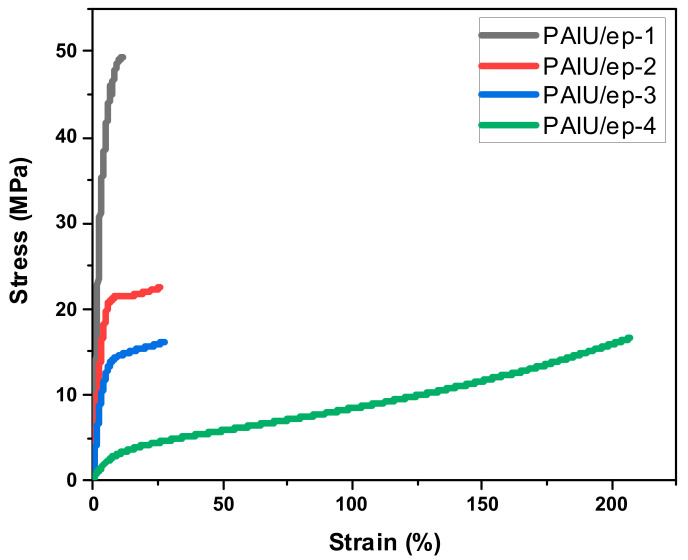
Stress-strain curves of PAIU/epoxy IPNs.

Having produced this compound, we prepared four types of PAIU/epoxy blends (PAIU/ep-1, PAIU/ep-2, PAIU/ep-3, and PAIU/ep-4) by varying the weight ratio of PAIU and an epoxy resin. The mixtures were cured using 2,4-diamino-6-[2-(2-methyl-1-imidazolyl)ethyl]-1,3,5-triazine (2MZ-A) as a curing agent. Given that the blend was cured to produce a three-dimensional polymer network in the presence of linear PAIU, it is assumed that final structure formed according to the blend mixture is a type of interpenetrating polymer network (IPN). The possible crosslinking between the amide groups of PAI and epoxide groups had been reported in the previous studies [39,40,41]. The effect of PAIU on the mechanical properties of PAIU/epoxy interpenetrating polymer networks (IPNs) was investigated through a tensile test. The PAIU/epoxy blends were poured into a silicone mold and cured at 150 °C for 120 min and 180 °C for 70 min sequentially to produce PAIU/epoxy IPN films with a dimension of 25 mm × 125 mm. Then, each film was cut into a dog-bone-shaped specimen using a press mold and subjected to a tensile test. The mechanical properties of the films are summarized in Table 2 and the stress-strain curves are shown in Figure 3. Although the tensile strength and Young’s modulus of the PAIU/epoxy IPN films decreased with increasing PAIU content, elongation at break increased, indicating that PAIU made the PAIU/epoxy IPN films ductile because of the soft segment of poly(tetrahydrofuran). Interestingly, all samples except PAIU/ep-4 showed a similar yield strain at about 10%, although their yield strengths differed. Once PAIU interpenetrated into a hard epoxy network with an epoxy content of more than 30 wt%, the PAIU/epoxy IPN films initially behaved like an epoxy thermosetting and deformed to resist breakage (plastic deformation) when the samples were further pulled. On the contrary, when the PAIU content reached 90 wt%, the PAIU/epoxy IPN film (PAIU/ep-4) became a stretchable elastomer with elongation at a break of >200%. This shows that the fabrication of IPNs by varying the ratio of two or more polymers is a useful way to adjust the properties of the resulting blend mixture [42].

**Figure 4:**
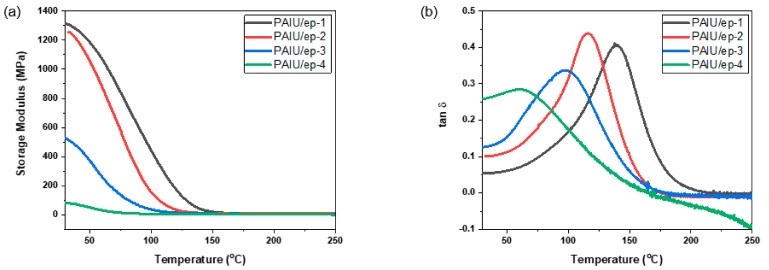
Temperature dependency of (**a**) storage modulus and (**b**) tan δ of PAIU/epoxy IPNs.

The effect of PAIU on the thermomechanical behavior of PAIU/epoxy IPNs was studied by dynamic mechanical analysis. For this purpose, the aforementioned films were cut into rectangular specimens (8 mm × 12 mm) using a CO_2_ laser. The temperature dependence of the storage modulus and loss factor (tan δ) was measured at temperatures ranging from 25 °C to 300 °C, as shown in Figure 4. The storage moduli of the PAIU/epoxy IPNs measured at 25 °C decreased with increasing PAIU content, which was in good agreement with Young’s modulus obtained from a tensile test. The Tg values of the PAIU/epoxy IPNs determined by the peak of the tan δ curve also decreased with increasing PAIU content because the soft segment of poly(tetrahydrofuran) promoted chain motion between molecules.

**Figure 5:**
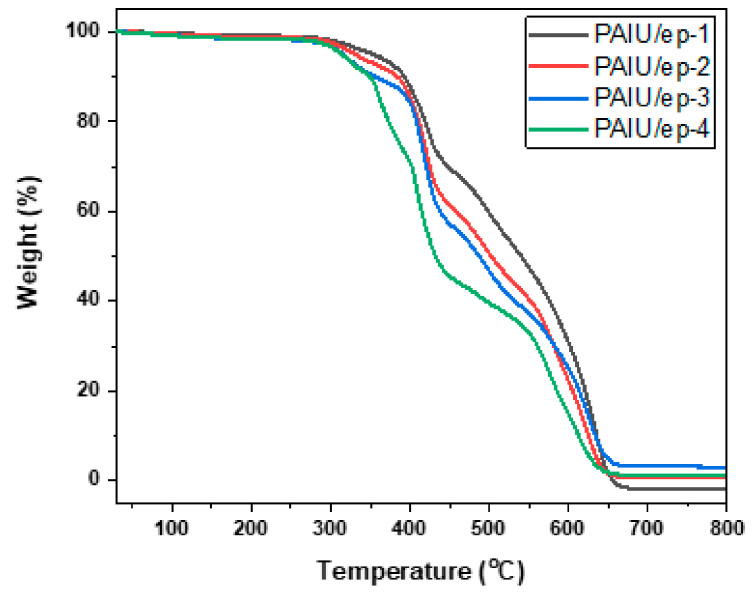
TGA thermographs of PAIU/epoxy IPNs.

Thermal stability is another crucial factor for the PAIU/epoxy IPNs to be applied as a coverlay that can remain intact to protect circuitry during soldering at approximately 300 °C. The thermal stability of the PAIU/epoxy IPNs was evaluated by thermogravimetric analysis (TGA) under air atmosphere, and the results are shown in Figure 5 and Table 3. All PAIU/epoxy IPNs were stable up to 300 °C with the weight loss of less than 5%, indicating that the PAIU/epoxy IPNs have sufficient heat resistance to be used as materials for a coverlay despite the presence of soft polyurethane.

The chemical resistance of the PAIU/epoxy IPNs was tested by immersing them in the acidic solution at 50 °C. After 24 h of immersion, the change in the weight of the PAIU/epoxy IPNs was measured, and the results are summarized in Table 4. All the PAIU/epoxy IPNs showed negligible weight reduction, indicating that they had excellent chemical resistance.

**Figure 6:**
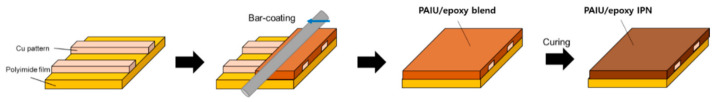
A schematic of the fabrication of a FPCB containing a PAIU/epoxy IPN as a coverlay.

Encouraged by the obtained results, we fabricated flexible printed circuit boards (FPCBs) using PAIU/epoxy IPNs to investigate the effect of PAIU on the mechanical durability of FPCBs. The PAIU/epoxy blends were bar-coated onto 30-μm-thick Cu patterns with line/space (1 mm/1 mm) and heated to produce the structure of PAIU/epoxy IPN layer/Cu patterns/PI film (Figure 6).

**Figure 7:**
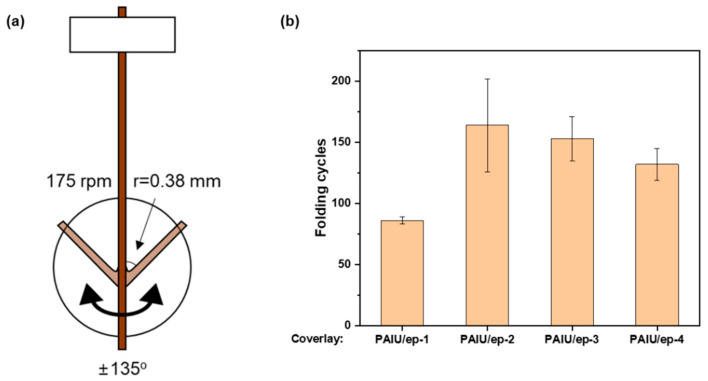
Measurements of folding endurance: (**a**) schematic of the folding endurance test; (**b**) the folding cycles measured until the Cu patterns on the FPCBs containing PAIU/epoxy IPNs as coverlays were ruptured.

The PAIU/epoxy IPN layer with the thickness of 25 μm serves as a coverlay to protect the Cu electrodes of the circuit board. Each FPCB was subject to an MIT folding test with a folding radius of 0.38 mm and a folding of 135 degrees (angle) (Figure 7). We recorded the number of folding cycles until a rupture of Cu patterns occurred, and the results are shown in Figure 7b. While the FPCB with PAIU/ep-1 was fractured after 86 folding cycles, other samples with higher PAIU content endured more than 130 folding cycles. Given that cracks were generated during a repetitive folding and propagated to the PAIU/epoxy IPNs, the soft PAIU was readily deformed to dissipate the applied energy; thus, increasing PAIU content affected the increase in resistance of the PAIU/epoxy IPNs to the fracture failure. The FPCB equipped with PAIU/ep-2 as a coverlay was more durable than the others and was stable up to 164 folding cycles, indicating that not only the elongation but also the modulus should be optimized to attain high-performance FPCBs [43,44,45,46,47,48].

## 4. Conclusions

In this work, a soft poly(amide-imide-urethane) was synthesized and used to fabricate flexible printed circuit boards. Thanks to the soft segment of poly(tetrahydrofuran), the incorporation of PAIU endowed epoxy resin with ductility. The elongation at break of the PAIU/epoxy IPNs increased with increasing PAIU content and reached 200% with a PAIU content of 90 wt%. Interestingly, these PAIU/epoxy IPNs showed high thermal stability and excellent chemical resistance despite the presence of the PU backbone. Next, flexible printed circuit boards containing the 25-μm-thick PAIU/epoxy IPNs as coverlays were fabricated. The softness of the PAIU/epoxy IPNs enhanced the mechanical durability of the FPCBs in an MIT folding test. We concluded that PAIU/ep-2 was the most suitable material for fabricating the coverlay of a FBPC because it outperformed the other materials considered in this study. Finally, the fabrication of durable coverlays through the simple coating of PAIU/epoxy blends is expected to be an effective way to realize thin high-performance FPCBs for flexible electronics.

## Figures and Tables

**Table 1:** Compositions of PAIU/epoxy blends.

	PAIU (g)	Epoxy (g)	2MZ-A (g)
PAIU/ep-1	30	70	0.7
PAIU/ep-2	50	50	0.5
PAIU/ep-3	70	30	0.3
PAIU/ep-4	90	10	0.1

**Table 2:** Mechanical properties and glass transition temperatures (Tgs) of PAIU/epoxy IPNs.

	PAIU/ep-1	PAIU/ep-2	PAIU/ep-3	PAIU/ep-4
Tensile strength (MPa)	50.0 ± 3.2	23.1 ± 1.3	16.8 ± 1.9	16.6 ± 3.7
Elongation at break (%)	11.4 ± 4.2	32.5 ± 9.0	33.6 ± 12.3	204.7 ± 38.6
Young’s modulus (MPa)	1120 ± 70	410 ± 30	320 ± 30	70/10
Tg (°C)	137.8	118.3	99.3	60.7

**Table 3:** TGA weight loss behavior of PAIU/epoxy IPN.

	Weight Loss at 300 °C	T_d,95_^a^ (°C)
PAIU/ep-1	1.88	354
PAIU/ep-2	2.33	331
PAIU/ep-3	3.32	316
PAIU/ep-4	3.36	314

^a^ temperature at 5% of the weight loss.

**Table 4:** Chemical resistance test results of PAIU/epoxy IPNs.

	PAIU/ep-1	PAIU/ep-2	PAIU/ep-3	PAIU/ep-4
Weight reduction (%)	0.62	0.36	0.59	1.20
